# Neuropathie périphérique sous Infliximab: étude d’une observation

**DOI:** 10.11604/pamj.2016.24.271.3498

**Published:** 2016-07-27

**Authors:** Ali Zinebi, Youssef Akhouad, Adil Rkiouak, Ahmed Reggad, Zohour Kasmy, Mostafa Boudlal, Monsef Rabhi, Khalid Ennibi, Jilali Chaari

**Affiliations:** 1 Service de Médecine Interne Hôpital Militaire Moulay Ismail I, Meknès, Maroc; 2 Service de Médecine, Interne A HMIM V, Rabat, Maroc

**Keywords:** Infliximab, anti TNF alpha, neuropathie périphérique, Infliximab, anti TNF alpha, peripheral Neuropathy, adverse effects, hemorrhagic colitis

## Abstract

Les traitements Anti TNF alpha sont de prescription de plus en plus large. Des événements secondaires multiples ont été rapportés ses dernières années, en particulier les neuropathies périphériques. Nous rapportons un cas de neuropathie axonale survenant trois mois après le début d’un traitement par Infliximab. Il s’agit d’une patiente âgée de 60 ans, suivie pour réctocolite hémorragique résistant au traitement et ayant nécessité un traitement par Infliximab. Trois mois après, la patiente présente un tableau de neuropathie axonale sensitive. Le bilan étiologique restait négatif et la réduction des doses s’est accompagnée d’une amélioration de la symptomatologie. Le délai entre l’instauration du traitement à base d’Infliximab et l’apparition des manifestations cliniques de même que l’amélioration après réduction des doses plaident en faveur de la responsabilité de l’Infliximab dans la survenue de la neuropathie sensitive. La prise en charge n’est pas standardisée et doit être discuté au cas par cas.

## Abstract (en)

Anti TNF alpha treatments are wide spectrum therapies. Multiple side effects have been reported in recent years, particularly peripheral neuropathies. We report a case of axonal neuropathy occurring three months after starting treatment with Infliximab. Our study focused on a 60-year old female patient treated for therapy-resistant hemorrhagic rectocolitis, requiring treatment with infliximab. Three months later, the patient had sensory axonal neuropathy. Etiologic assessment remained negative and dose reduction was accompanied by an improvement in symptoms. The time between initiation of treatment with Infliximab and the onset of clinical manifestations as well as improvement after dose reduction advocate the responsibility of infliximab in the occurrence of sensory neuropathy. Its management is not standardized and should be discussed case by case.

## Introduction

Le TNF alpha est une des principales cytokines pro-inflammatoires de l’organisme dont les principales sources cellulaires sont représentées par les monocytes ou les macrophages et les lymphocytes T. Cette cytokine joue un rôle physiopathologique dans de nombreuses maladies inflammatoires où l’efficacité des agents anti-TNF alpha a été bien démontrée. Leur introduction dans l’arsenal thérapeutique des rhumatismes inflammatoires et des maladies inflammatoires chroniques de l’intestin a modifié leur pronostic. Il peut s’agir d’une part d’anticorps monoclonaux chimériques ou humanisés (Infliximab ou Adalimumab) et d’autre part, d’inhibiteur compétitif de la fixation du TNF alpha à ses récepteurs (Etanercept). Les neuropathies périphériques induites par les anti-TNF alpha sont rares et hétérogènes. Des polyradiculonévrites aiguës ou chroniques, des mononeuropathies multiples et des polyneuropathies ont été décrites dans la littérature. Nous rapportons un nouveau cas de polyneuropathie axonale sensitive survenant sous Infliximab chez une patiente suivie pour une réctocolite hémorragique sévère.

## Patient et observation

Une femme de 60 ans signalait l’installation rapidement progressive ; aux extrémités des quatre membres ; de fourmillement, de paresthésie à type de picotements avec des dysesthésies sous forme de sensations désagréables et inconfortables. La patiente décrit également des douleurs brûlantes, surtout aux pieds.

Dans ses antécédents, on ne retrouvait pas d’affections neurologiques et n’était pas connue porteuse d’hépatopathie virale C, mais une rectocolite hémorragique avec une polyarthrite périphérique invalidante. Un traitement par l’Infliximab, à raison de 5mg/ kg/ cure, était instauré devant la non amélioration de la symptomatologie sous sulfasalazine et corticoïde. Trois mois après, l’évolution était favorable sur le plan digestif et articulaire avec apparition de signes clinique évoquant une neuropathie périphérique sans xérostomie ni xérophtalmie. La patiente ne rapportait pas de changements dans la transpiration, de modifications vasomotrices cutanées, de dysfonctionnements vésicaux, ou de symptômes d’hypotension orthostatique L’examen clinique ne retrouvait pas d’amyotrophie ni déficit moteur mais une hypoesthésie symétrique en gant et en chaussette avec une abolition des réflexes achilléens.

L’éléctroneuromyogramme retrouvait un aspect de poly neuropathie sensitive de type axonale aux membres inférieurs et un aspect du syndrome du canal carpien bilatéral (Tableau 1) avec une vitesse de conduction sensitive (VCS) au niveau du nerf médian droit de 45 m/s, médian gauche de 46 m/s, sural droit à 33 m/s) Sur le plan biologique, on ne retrouvait pas de syndrome inflammatoire ni déficit en vitamine B12, l’hémogramme était normal et les sérologies virales (VIH, HCV) sont négatives. On n’objectivait pas de syndrome de mal absorption, la glycémie était normale de même que le bilan thyroïdien. Le bilan immunologique, notamment la recherche d’AAN et d’ANCA, était négatif. Devant la négativité du bilan étiologique, l’anti TNF alpha (Infliximab) a été retenue comme étant la cause la plus probable du fait de sa récente introduction chez notre patiente.

**Tableau 1:** Évolution des examens électrophysiologiques de la patiente

	ENMG DU 17/08/2012				ENMG DU 17/06/2013			
	VCM (m/s)	Latence distale (ms)	Amplitude D (mV)	Onde F	VCM (m/s)	Latence distale (ms)	Amplitude D (mV)	Onde F
**Conductions motrices**								
Médian droit	61	4.1	18	26	58	3.4	22	26
Médian gauche	56	3.8	16	52.6				
Cubital droit	68	3	11	25	59	2.2	13	26
Cubital gauche	53	3.4	14	25.2				
SPE droit	46	4.4	4.7	47	47	3.3	8	47
SPE gauche	50	4.9	2.1					
SPI droit	46	5.5	9	46	46	5.3	11	46
SPI gauche	45	5	14	48				
**Conductions sensitives**								
Médian droit	45	3.1	27		65	2.2	28	
Médian gauche	46	3	36					
Cubital droit	46	3	38		66	2.1	30	
Cubital gauche	48	2.9	27					
Sural droit	33	3	3		48	1.3	12	
Cubital gauche	40	2.5	6					

L’Infliximab n’a pu être arrêté en raison de la gravité de la maladie intestinale et une réduction de la dose à 3mg/Kg/ cure a été instaurée. L’évolution était marquée par une amélioration progressive mais franche de la symptomatologie. L’ éléctroneuromyogramme réalisé 10 mois après la réduction des doses (Tableau 1) a montré une régression totale de la polyneuropathie et du syndrome de canal carpien : VCS est de 65 m/s au nerf médian droit et de 48 m/s au nerf sural droit.

## Discussion

La survenue d’effets indésirables neurologiques périphériques (Tableau 2) sous anti-TNF alpha est une problématique devant laquelle les prescripteurs risquent d’être de plus en plus souvent confrontés, en raison du recule à l’utilisation grandissante de cette famille thérapeutique et aussi grâce à une meilleure sensibilisation à ces effets secondaires.

**Tableau 2:** Caractéristiques des neuropathies périphériques survenant au court du traitement par les antagonistes des TNF alpha

Auteur (référence)	Nombre (âge (an), sexe)	Type de neuropathie	Produit	Affection sous jacente	Délais	Décision	Evolution
Notre observation	1 (60, F)	Polyneuropathie sensitive de type axonale	Infliximab	Récto Colite Hémorragique	3 mois	Réduire les doses à 3mg/kg	favorable
Singer et Al(3)	(28, F)	PRN	Infliximab	Crohn	2 ans	Arrêt +corticoïde	Sans amélioration initialement puis spontanément favorable en 5 semaines
	(45, F)	PRN	Infliximab	Colite microscopique	3 mois		
Rodriguez-Escalera (4)	1 (34, F)	Neuropathies motrices multifocales	Infliximab	Polyarthrite rhumatoide	4 mois	Arrêt plus immunoglobuline	Favorable
Cocito (5)	1(40, F)	Neuropathies motrices multifocales	INFLIXIMAB	Polyarthrite rhumatoide	9 mois	Arrêt plus immunoglobuline	Favorable
Hamon M.A (14)	1 (39, F)	Polyneuropathie sensitivo-motrice démyélinisante	Adalimumab	Polyarthrite rhumatoide	1 mois après réintroduction	Immunoglobuline plus corticoïde	Inefficacité initialement puis amélioration secondaire
Richette (6)	(41, F)	Mononeuropathie Sensitive	Infliximab	Polyarthrite rhumatoide	6 mois	Arrêt + corticoïde	Favorable
	(48, F)	Mononeuropathie multiple Sensitive	Infliximab	PR/vascularite	8 heurs	Arrêt + corticoïde + cyclophosphamide	Amélioration légère
Richez (8)	(73, F)	Polyradiculonévrite chronique	Etanercept	Polyarthrite rhumatoide	17 mois	Arrêt	Favorable
	(47, H)	Polyradiculonévrite chronique	Infiximab	Spondylarthrite ankylosante	4 mois	Arrêt	Favorable
Tektonidou (9)	(60, H)	Neuropathie motrice multifocale	Infliximab	Polyarthrite rhumatoide	5 mois	Immunoglobuline	Favorable
	(56, F)	Polyneuropathie axonale sensitive	Infliximab	Polyarthrite rhumatoide	3 mois	Immunoglobuline	Favorable

Il peut s’agir de neuropathies démyélinisantes, de neu¬ropathies périphériques ou de vascularites. Ces manifestations pourraient être isolées ou parfois entrer dans le cadre d’une pathologie dysimmunitaire systémique induite. L’estimation de leur incidence est difficile et le délai de survenue est souvent inférieur à deux ans après le début de l’exposition aux anti-TNF alpha [1]. Dans notre cas, les manifestations ont été constatées après la troisième cure soit au troisième mois. Il est de 4 mois pour les patients de Singer et al. (2004), [2] et de Rodriguez-Escalera et al. (2005) [3], 6 mois pour ceux de Cocito et al. (2005) [4] et Richette et al. (2004) [5].

Les neuropathies périphériques peuvent être de différentes types: il peut s’agir de mononeuropathie sensitive comme c’est les cas rapportés par Richette et al, (2004) [5] révélant une vascularite nécrosante après introduction d’infliximab, ou de polyradiculonévrite chronique idiopathique inflammatoire après initiation d’un traitement par anti TNF alpha (Richez et al., 2005) [6]. Tektonidou (2006) [7] a décrit une polyneuropathie axonale sensitive après introduction d’infliximab comme c’est le cas chez notre patiente. Certaines observations se particularisaient par le fait qu’il existait une autre étiologie possible pour ces neuropathies : existence d’une maladie de Crohn et d’une colite microscopique pour les patientes de Singer et al. (2004) [2], et l’existence d’une hépatite C et d’une cryoglobuline pour la patiente de Rodriguez-Escalera et al. (2005) [3]. Il est possible que la présence de la maladie intestinale inflammatoire auto-immune, qui elle-même peut être associée à la multinévrite, facilite la réaction à l´Infliximab.

Le délais d’apparition des symptômes et la bonne tolérance lors des premières perfusions, de même que le caractère diffus et homogène des anomalies de conduction et l’inefficacité des traitements par immunoglobulines polyvalentes et corticoïdes, témoignent plutôt de la toxicité directe de l’Infliximab bien qu’un mécanisme immunologique favorisant est possible étant donné l’incrimination de la molécule comme inductrice de production d’anticorps anti nucléaires et la réaction immunologique à cet anticorps monoclonal peut induire une neuropathie auto-immune.

Bien que les anti-TNF soient tous classés dans la même catégorie, le risque de neuropathie et d’autres complications neurologiques varie d’un anti-TNF à l’autre (Ramos-Casals M, 2007) [8]. A notre connaissance, très peu de cas de neuropathie induite par l’Adalimumab ont été décrits à ce jour. Ce fait pourrait traduire une moindre réactivité immunologique croisée avec l’Adalimumab qu’avec l’Infliximab. Néanmoins, les données publiées restent rares et cette hypothèse doit donc être envisagée avec prudence.

Le degré de causalité entre l´événement constaté et la prise du médicament a été établi en utilisant la méthode d’imputabilité française (imputabilité intrinsèque, imputabilité extrinsèque). Dans notre observation, l’application des scores d’imputabilité retrouve un score sémiologique plausible (S2) et un score chronologique vraisemblable (C3). Le score d’imputabilité intrinsèque est par conséquent I3 donc l’imputabilité est vraisemblable. Quant à l’imputabilité extrinsèque, elle est classée B1 L’évolution est habituellement toujours favorable après arrêt du traitement : soit spontanément pour les cas de Singer et al. (2004) [2] et de Richez et al. (2005) [6]; soit après perfusions d’immunoglobulines dans les cas de Rodriguez-Escalera et al. (2005) [3] et de Cocito et al. (2005) [4]; ou perfusion de corticoïdes et de cyclophosphamide pour les cas de Richette et al. (2004) [5], et après perfusions d’immunoglobulines intraveineuses pour les 2 patients de Tektonidou et al. (2006) [7]. Pour la patiente de Hamon [9], les perfusions d’immunoglobulines et de solumédrol se sont révélées inefficaces et une amélioration spontanée n’a été constatée que secondairement.

Dans l’étude de Stubgen et al [10], une prise en charge spécifique de la neuropathie par corticoïdes et immunoglobulines intraveineuses était le plus souvent nécessaire malgré une amélioration spontanée de la symptomatologie à l’arrêt des anti TNF alpha. D’autres auteurs suggèrent une évolution à long terme aléatoire et estiment que la décision de poursuivre le traitement par anti TNF alpha doit être discutée au cas par cas. Le degré de récupération et l’existence de séquelles après arrêt de l’anti-TNF alpha ne sont pas bien connus, les durées de suivi rapportées dans la littérature étant trop courtes pour pouvoir en juger. Chez notre patiente, l’évolution est partiellement favorable avec régression presque complète des symptômes après trois mois de réduction de la posologie et une récupération électrique complète au 10^ème^ mois.

## Conclusion

**Figure 1:**
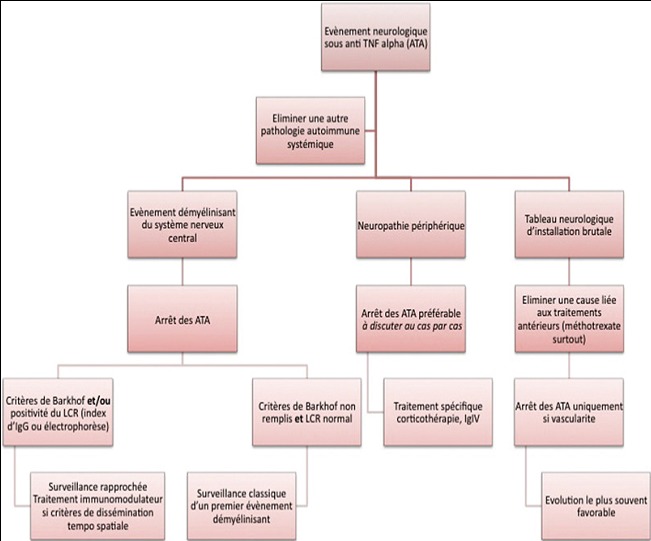
Proposition de prise en charge standardisée des évènements neurologiques survenant sous anti-TNF alpha (D’après Cohen M, Baldin B, Thomas P, Lebrun C. Evènements neurologiques sous traitement par anti-TNF alpha revue neurologique 168 (2012) 33-39)

L’Infliximab est l’un des médicaments dont l’efficacité est incontestable dans les maladies inflammatoires chroniques de l’intestin et les rhumatismes inflammatoires. En cas de survenue d’une neuropathie périphérique, les données de la littérature incitent à discuter au cas par cas la poursuite du traitement et l’instauration éventuelle d’un traitement spécifique de la neuropathie (Figure 1).
